# Disparity in refuge: the plight of international students seeking health and education in the midst of the Ukrainian war

**DOI:** 10.1007/s40037-022-00722-y

**Published:** 2022-07-19

**Authors:** Wireko Andrew Awuah, Aashna Mehta, Rohan Yarlagadda, Jyi Cheng Ng, Toufik Abdul-Rahman

**Affiliations:** 1 Medical Institute, Sumy State University, Sumy, Ukraine; 2 Faculty of Medicine, University of Debrecen, Debrecen, Hungary; 3 Rowan University School of Osteopathic Medicine, Stratford, NJ USA; 4 Faculty of Medicine and Health Sciences, University of Putra Malaysia, Serdang, Malaysia

To the Editor,

One day a bustling country, the next day a war zone. The Russian invasion of Ukraine was a terrifying fear that shockingly turned to reality. The war has significantly disrupted Ukrainian life and ongoing coverage has revealed the grim aftermath of the unfolding violence. As much of the world stands in solidarity with Ukrainians, stories of unequal treatment of international students attempting to flee have begun to surface.

There are over 76,000 international students from 155 countries studying in Ukraine, the majority of whom are enrolled in Medicine and Health Sciences [1]. According to Ukraine’s Ministry of Education and Science, in late 2020 international students contributed a total of $ 570 m annually in national revenue, representing a significant contribution to the country’s economy [1]. International students also have a notable cultural impact by introducing a plethora of various languages and customs that deepen Ukrainian diversity. However, since the beginning of the war, it appears as though international students have been treated unfairly. These refugees were being stopped from boarding trains, pushed off buses and trains, and forced to walk those long distances [2]. At the border, Ukrainian natives were prioritized to cross while international residents were segregated, forced to wait or even denied at the border. Meanwhile, some international groups that chose to remain in some EU member countries were detained and were threatened with deportation despite the ongoing crises. Universities around the world have clearly stated their intention to assist Ukrainian refugees, but the displaced international students seeking similar assistance were told that “this opportunity is only for Ukrainian citizens” [3]. This has forced a significant disruption to their education with sparingly few avenues to overcome it. One must also consider the war’s psychological turmoil being exacerbated with such discriminatory actions against international residents in Ukraine.

The war has subjected Ukrainian residents to frightening realities in which lives are in danger and access to vital resources such as food, water and sanitation is severely limited. It stands to emphasize that all Ukrainian residents, native or international, have been subjected to the same horrors of war and therefore deserve equal access to humanitarian aid, corridors, and refuge in neighboring countries. It is in our best interests as a global society to put aside our differences and work together to provide relief and support to all refugees in order to improve war outcomes.
